# From skin clearance to psychological wellbeing: real-world outcomes of biologic therapy in psoriasis

**DOI:** 10.3389/fpsyg.2026.1735777

**Published:** 2026-02-13

**Authors:** Francesco Cuniberti, Marco Miniotti, Mariagiulia Bailon, Luca Mastorino, Michela Ortoncelli, Angelo Picardi, Simone Ribero, Paolo Leombruni

**Affiliations:** 1 Clinical Psychology Unit, “Rita Levi Montalcini” Department of Neuroscience, University of Turin, Turin, Italy; 2 Dermatology Clinic, Department of Medical Sciences, University of Turin, Turin, Italy; 3 Center for Behavioral Sciences and Mental Health, Italian National Institute of Health, Rome, Italy

**Keywords:** biologic therapy, perceived stress, psoriasis, psychological distress, quality of life, wellbeing, WHOQOL-BREF

## Abstract

**Background:**

Psoriasis impacts psychological and quality-of-life (QoL). While biologic therapies demonstrated robust efficacy in reducing skin lesions, their broader psychosocial impact remains underexplored in real-world settings. This ambispective observational cohort study evaluated clinical, psychological, and health-related QoL (HRQoL) outcomes of biologic therapy in patients with moderate-to-severe plaque psoriasis treated in routine dermatological practice.

**Methods:**

A total of 133 patients undergoing biologic therapy at a referral center in Northern Italy were assessed. Baseline data (T0) were retrospectively extracted from medical records, while 6-month follow-up assessments (T1) were conducted prospectively. Clinical severity (PASI), depression (PHQ-9, BSI-18), anxiety and somatization (BSI-18), perceived stress (PSS), dermatology-specific QoL (DLQI), and general HRQoL (WHOQOL-BREF) were evaluated. Regression models identified baseline predictors of T1 psychological and QoL outcomes.

**Results:**

At follow-up, patients reported significant improvements in psychological wellbeing and QoL. PHQ-9 scores decreased markedly (*p* < 0.001), with the prevalence of moderate-to-severe depressive symptoms dropping from 28.6% to 5.3%. Substantial perceived stress (PSS ≥ 27) declined from 15.0% to 1.5%. DLQI scores showed a large effect size (*p* < 0.001), with 89.5% reporting minimal impact (DLQI 0–1) at follow-up. Regression analyses identified baseline psychological symptoms as the strongest predictors of follow-up psychological and QoL outcomes. Additional predictors included PASI, female sex, psychiatric comorbidity, and previous biologic therapy. At T1, 77.4% achieved complete skin clearance (PASI 100).

**Conclusion:**

Biologic therapies confer multidimensional benefits in moderate-to-severe psoriasis, extending beyond skin clearance to substantial reductions in psychological distress and improvements in QoL. These findings support a patient-centered model integrating dermatologic and mental health care.

## 1 Introduction

Psoriasis is a chronic, immune-mediated inflammatory skin disorder characterized by erythematous, thickened, scaly, pruritic, and often painful plaques, most commonly affecting the elbows, knees, scalp, and trunk (Griffiths et al., 2021; Taliercio and Lebwohl, 2024). It affects approximately 125 million people globally (Parisi et al., 2020), with a prevalence ranging from 1.8% to 4.8% in Italy (Gianfredi et al., 2022). Beyond its visible cutaneous manifestations, psoriasis is a systemic disease with well-documented psychiatric and other medical comorbidities (Hedemann et al., 2022; Jiang et al., 2023; Cuniberti et al., 2024; Taliercio and Lebwohl, 2024). The psychosocial burden of psoriasis is substantial and often underrecognized in clinical settings, as shown by recent clinic-based studies reporting high levels of anxiety, depression, stigma, and impaired wellbeing among dermatology outpatients (Sondermann et al., 2021; Silva et al., 2025). In addition to chronic pain, disfigurement, and functional disability, patients with psoriasis experience disproportionately high rates of mental health conditions such as depression, anxiety, sleep disturbances, substance use, and social withdrawal (Gondo et al., 2021; Hedemann et al., 2022; Robinson et al., 2023; Taliercio and Lebwohl, 2024). Psoriasis is also linked to increased risk of suicidal ideation and behavior, particularly in severe cases or with lesions on stigmatized areas such as the face or genitals (Picardi et al., 2006, 2013; Rivers and Mar, 2023; Salari et al., 2024; Taliercio and Lebwohl, 2024). Despite increasing clinical awareness, psychiatric comorbidities often remain underdiagnosed and untreated in dermatological practice, highlighting the need for more integrated and multidisciplinary care models (Strober et al., 2018; Hedemann et al., 2022; Liu L. et al., 2023; Wang et al., 2023). The bidirectional relationship between psoriasis and psychological distress creates a self-perpetuating cycle: psychological stress exacerbates cutaneous inflammation, while disease flares and persistent symptoms negatively affect self-esteem and psychological wellbeing (Basavaraj et al., 2010; Gianfredi et al., 2022; Liu L. et al., 2023; Taliercio and Lebwohl, 2024; Wintermann et al., 2024). Pro-inflammatory cytokines—such as TNF-α, IL-6, and IL-17—have been implicated in the pathophysiology of psoriasis, as well as mood and anxiety disorders. This overlap suggests the presence of shared neuroimmune mechanisms that may underlie their frequent co-occurrence (de Alcantara et al., 2021; Hedemann et al., 2022; Jiang et al., 2023). Over the past decade, the therapeutic landscape for moderate-to-severe psoriasis has evolved substantially with the introduction of targeted biologic and small-molecule therapies. In parallel, growing evidence has highlighted their potential benefits beyond skin clearance, particularly in improving psychological wellbeing and health-related QoL (Martínez-Ortega et al., 2019; Leman et al., 2020; Reich et al., 2021; Rzeszutek et al., 2021; Liu J. et al., 2023; Cuniberti et al., 2024). Evidence from RCTs, observational studies, and real-world cohorts suggests reductions in depressive and anxiety symptoms among patients treated with TNF-α inhibitors (e.g., adalimumab, infliximab), IL-17 inhibitors (e.g., secukinumab), and IL-23 inhibitors (e.g., guselkumab) (Strober et al., 2018; Reich et al., 2021; Liu L. et al., 2023; Liu J. et al., 2023; Liu et al., 2024; Mastorino et al., 2024). These improvements appear to be mediated by both skin clearance and systemic immunomodulation, with some studies suggesting direct effects on cytokine pathways involved in mood regulations (de Alcantara et al., 2021; Hedemann et al., 2022; Mohd Noor et al., 2022). However, the current literature presents several limitations (Wang et al., 2024). Many studies lack long-term follow-up, use small or selective samples, or do not include standardized psychiatric assessments (Strober et al., 2018, 2024; Hedemann et al., 2022; Liu J. et al., 2023; Wang et al., 2024). The heterogeneity in tools, endpoints, and study populations also complicates interpretation. As a result, further research is needed to better understand the psychological impact of biologics and to clarify whether the observed benefits are sustained in real-world practice (Strober et al., 2018, 2024; Wang et al., 2024).

This ambispective, observational real-world study aimed to investigate clinical, psychological, and QoL outcomes in patients with moderate-to-severe plaque psoriasis treated with biologic agents in a routine clinical setting.

Specifically, the objectives were to:

1. Assess changes in psoriasis severity, psychological symptoms (depression, anxiety, somatization), perceived stress, and dermatology-specific and general HRQoL between baseline (T0, retrospectively collected) and six-month follow-up (T1, prospectively assessed).
2. Estimate the prevalence and severity of depressive symptoms, stress, and QoL impairment at both time points using validated cut-offs.
3. Identify baseline demographic, clinical, and psychological predictors of psychological distress and QoL outcomes at T1 via multivariable regression.

## 2 Materials and methods

### 2.1 Study design, procedures, and participants

This ambispective observational cohort study was conducted at the Dermatology Clinic of the “*Città della Salute e della Scienza*” University Hospital in Turin, an academic referral center for psoriasis in Northern Italy. Consecutive adult patients (≥18 years) with a diagnosis of moderate-to-severe plaque psoriasis who had initiated biologic therapy during routine outpatient visits between January 2023 and January 2025 were eligible. Patients receiving conventional systemic treatments were excluded. Patients receiving conventional systemic treatments were excluded. This criterion reflects the therapeutic pathway adopted in Italy for moderate-to-severe psoriasis. Eligibility for biologic therapy is nationally regulated and restricted to patients within this severity range; consequently, only individuals with moderate-to-severe disease are routinely referred to tertiary care centers for treatment initiation. The Dermatology Clinic of the Turin University Hospital serves as a tertiary referral hub, typically receiving patients who have already been pretreated with topical agents and at least one conventional systemic therapy (e.g., methotrexate or cyclosporine) in primary care, private dermatology settings, or peripheral hospitals. As mandated by national regulations, traditional systemic therapies must be discontinued upon initiation of a biologic agent, which justifies the exclusion of patients currently receiving such treatments. Recruitment was conducted prospectively among patients attending their scheduled 6-month follow-up dermatology visit (T1) after biologic therapy initiation. The 6-month visit corresponds to the first standardized clinical reassessment after biologic initiation in our center. This timing is consistent with international guidelines and regulatory trials, which commonly use the 16-week window to define primary treatment failure based on PASI outcomes. Although biologic classes differ in their time-to-peak response— with IL-17 inhibitors reaching maximal efficacy earlier and IL-23 inhibitors showing more gradual improvement— the six-month mark ensures adequate therapeutic exposure across all agents. Accordingly, a 6-month assessment provides a clinically and methodologically consistent window in which the vast majority of biologics have expressed their full therapeutic potential, allowing meaningful comparisons of clinical, psychological, and QoL outcomes at T1. Only patients who provided written informed consent were included. At the T1 visit, participants underwent a structured clinical and psychological evaluation conducted by a psychiatrist and a licensed psychologist with expertise in psychodermatology. Psychiatric comorbidities were identified using a DSM-5–based interview, integrating patient history and medical records; baseline (T0) conditions were recorded only if confirmed at T1. Socioeconomic status was self-reported using a single-item measure reflecting perceived income, occupational status, and financial strain. Baseline data (T0), including disease history, Psoriasis Area and Severity Index (PASI) scores, and psychometric measures routinely administered at treatment initiation, were retrospectively extracted from the center’s electronic medical records. Because T0 data were retrospectively extracted from electronic records at the time of biologic initiation, the exact timing of baseline documentation differed slightly across participants. Although this variability reflects real-world clinical practice, it could introduce heterogeneity in baseline values that could not be statistically controlled. The study protocol was approved by the local ethics committee.

### 2.2 Clinical, psychological, and QoL measures

Psoriasis severity was assessed using the Psoriasis Area and Severity Index (PASI) (Fredriksson and Pettersson, 1978), with treatment response expressed as percent reduction from baseline and PASI 75, 90, and 100 as key outcomes. Depressive symptoms were evaluated with the Patient Health Questionnaire-9 (PHQ-9) (Spitzer et al., 1999; Picardi et al., 2005), perceived stress with Perceived Stress Scale (PSS) (Cohen et al., 1983) and global psychological distress with the Brief Symptom Inventory-18 (BSI-18) (Derogatis and Melisaratos, 1983), which includes depression, anxiety, and somatization subscale. Dermatology-specific QoL was assessed with the Dermatology Life Quality Index (DLQI) (Finlay and Khan, 1994; Mazzotti et al., 2003, 2005), and global QoL with the World Health Organization Quality of Life – BREF (WHOQOL-BREF) (THE WHOQOL GROUP, 1998). Higher scores indicate greater symptom severity or perceived stress; for QoL measures, higher DLQI indicates worse QoL, whereas higher WHOQOL-BREF indicates better QoL. Detailed descriptions of scoring ranges, cut-offs, and interpretation are provided in Supplementary Table 1. In addition, internal consistency for all psychometric instruments was assessed using Cronbach’s alpha. Symptom scales demonstrated acceptable to excellent reliability (α = 0.79–0.95), whereas lower values for WHOQOL-BREF domains were expected due to their heterogeneous and multidimensional nature. Cronbach’s alpha coefficients for each instrument and domain are reported in Supplementary Table 4.

### 2.3 Statistical analyses

Analyses were conducted using IBM^®^ SPSS^®^ Statistics v30 (IBM Corp., Armonk, NY, United States). Categorical variables were summarized as counts/percentages and continuous variables as means/SDs, given the robustness of parametric tests in large samples. Normality of paired differences was assessed via Q-Q plots and histograms supporting the use of parametric procedures. Missing data were handled via listwise deletion for inferential and pairwise deletion for descriptive analyses. Paired-sample *t*-tests compared baseline (T0, retrospectively collected) and follow-up (T1, prospectively assessed) scores on clinical, psychological, and QoL measures. Effect sizes (Cohen’s d) were computed. Baseline predictors of T1 outcomes were explored using stepwise multiple linear regression (entry: *p* ≤ 0.05; removal: *p* ≥ 0.10) to identify relevant predictors while limiting overfitting. Independent variables included sociodemographic, clinical, and baseline psychometric measures (full list in Supplementary Table 2); categorical variables were dummy-coded. Baseline PASI score was treated as a continuous variable and entered as a linear predictor in all regression models. Supplementary Table 2 summarizes all candidate predictors entered into the regression models, detailing variable definitions, coding procedures, and inclusion criteria across sociodemographic, clinical, and baseline psychometric domains, thereby providing full transparency regarding predictor operationalization. For multi-level predictors, additional simple regressions clarified the direction of associations. For each model, F-statistic, *p*-value, adjusted R^2^, and *p*-values of significant predictors were reported; standardized β coefficients are provided in Supplementary Table 3. All regression assumptions were satisfied, including normality, linearity, and homoscedasticity of residuals. No influential outliers were detected (Cook’s distance values < 1), and multicollinearity was not a concern (VIF < 1.5; Tolerance > 0.70).

## 3 Results

### 3.1 Sociodemographic and clinical characteristics

This study included 133 participants. The mean age was 51.95 ± 14.9 years, and 63.2% were male (*n* = 84). The mean age at disease onset was 28.78 ± 12.28 years. Relevant medical comorbidities were reported in 45.1% of patients (*n* = 60). All participants received a biologic agent: anti–IL-17 (52.63%, *n* = 70), followed by anti–IL-23 (31.58%, *n* = 42), anti–TNFα (9.77%, *n* = 13), and anti–IL-12/23 (6.02%, *n* = 8). No serious adverse events resulting in treatment discontinuation were reported during the observation period. Full details are presented in Table 1.

**TABLE 1:** Sample characteristics.

Variable	Category	*N* (%)
Sex	Male	84 (63.2%)
Psoriatic arthritis	Present	23 (17.4%)
Medical comorbidities	Present	60 (45.1%)
	Hypertension	30 (22.6%)
	Obesity (BMI ≥ 30 kg/m^2^)	22 (16.5%)
	Diabetes	12 (9.0%)
Psychiatric comorbidities	Present	8 (6.0%)
	Anxiety disorders	3 (2.3%)
	Major depressive disorders	2 (1.5%)
	Bipolar disorders	2 (1.5%)
Marital status	Single	23 (17.3%)
	Married or cohabiting	91 (68.4%)
	Separated/divorced/ widowed	19 (14.3%)
Educational level	Primary/secondary school	57 (42.9%)
	High school diploma	49 (36.8%)
	University degree	24 (18.0%)
	Postgraduate	3 (2.3%)
Living arrangement	Alone	27 (20.3%)
	With partner or family	104 (78.2%)
	Other	2 (1.5%)
Economic status	Low	30 (22.6%)
	Medium	87 (65.4%)
	High	16 (12.0%)
Employment status	Employed	76 (57.1%)
	Unemployed	54 (40.6%)
	Student	3 (2.3%)
Previous standard therapy	Received	124 (93.2%)
Previous biologic therapy	Received (single or multiple)	35 (26.9%)
Biologic class (current)	Anti-IL-17	70 (52.63%)
	Anti-IL-23	42 (31.58%)
	Anti-TNFα	13 (9.77%)
	Anti-IL-12/23	8 (6.02%)
PASI response at T1	PASI 100	103 (77.4%)
	PASI 90	24 (18.0%)
	PASI 75	4 (3.0%)
	PASI < 75	2 (1.6%)
**Continuous variables**	**Mean ± SD**	
Age (years)	51.95 ± 14.9	
Age at disease onset (years)	28.78 ± 12.28	
BMI (kg/m^2^)	25.81 ± 4.24	
**PASI**		
Baseline (T0)	15.59 ± 6.08	
Follow-up (T1)	0.38 ± 1.40	
PASI reduction (%)	97.72 ± 7.08	

Data are presented as *n (%)* for categorical variables and mean ± SD for continuous variables. BMI, body mass index; PASI, Psoriasis Area and Severity Index.

### 3.2 Comparisons between baseline and follow-up measures

Comparative analyses between baseline (T0, retrospectively collected) and 6-month clinical re-evaluation (T1, prospectively assessed) indicated statistically significant improvements across all clinical, psychological, and quality-of-life outcomes (see Table 2 for details). PASI scores showed a marked reduction (t = 28.88, *p* < 0.001, d = 2.50), with 3.0% of participants achieving PASI 75 (*n* = 4), 18.0% achieving PASI 90 (*n* = 24), and 77.4% achieving complete skin clearance (PASI 100, *n* = 103) at T1. PHQ-9 scores significantly improved (t = 6.67, *p* < 0.001, d = 0.58), with notable reductions in the frequency of depressive severity: at T0, 9.8% of participants reported mild, 18.8% moderate, 8.3% moderately severe, and 1.5% severe depression; at T1, these frequencies decreased to 4.5%, 3.0%, 1.5%, and 0.8%, respectively. PSS scores significantly decreased (t = 12.64, *p* < 0.001, d = 1.10), with substantial perceived stress (PSS ≥ 27; high range) declining from 15.0% to 1.5%, while the overall distribution shifted from low 12.0%/moderate 72.9%/high 15.0% at baseline to 61.7%/36.8%/1.5% at follow-up. The BSI-18 Global Severity Index (GSI) significantly declined (t = 8.09, *p* < 0.001, d = 0.70), with specific improvements in anxiety (t = 9.54, *p* < 0.001, d = 0.82), somatization (t = 5.84, *p* < 0.001, d = 0.51), and depression (t = 3.69, *p* < 0.001, d = 0.32) subscales. DLQI scores showed a substantial reduction (t = 33.84, *p* < 0.001, d = 2.93). At baseline, 21.1% of participants reported moderate, 68.4% very large, and 10.5% extremely large impact of psoriasis on QoL, whereas at follow-up, 89.5% reported a small effect, 9.0% a moderate effect, and only 0.8% a large or very large effect. Finally, all WHOQOL-BREF domains demonstrated significant improvements from baseline to follow-up. Scores increased in the physical health domain (t = −14.78, *p* < 0.001, d = −1.28), the psychological domain (t = −24.36, *p* < 0.001, d = −2.10), the social relationships domain (t = −13.00, *p* < 0.001, d = −1.13), and the environmental quality domain (t = −14.16, *p* < 0.001, d = −1.23), indicating broad and substantial enhancements in patients’ perceived QoL across multiple dimensions. At baseline, women showed significantly higher perceived stress (PSS-10; *p* = 0.048) and lower psychological wellbeing (WHOQOL-BREF psychological domain; *p* = 0.036) compared with men. No significant sex differences were observed for depressive symptoms, anxiety, somatization, dermatology-specific QoL, or the remaining WHOQOL-BREF domains (Supplementary Table 5).

**TABLE 2:** Paired-sample *t*-test results for T0 vs T1 comparisons.

Outcome measure	T0 (mean ± SD)	T1 (mean ± DS)	t	*P*-value	Effect size (r)	Direction
PASI	15.59 ± 6.08	0.38 ± 1.40	28.88	<0.001	2.50	↓
PHQ-9	5.20 ± 5.82	1.97 ± 3.36	6.67	<0.001	0.58	↓
PSS	20.74 ± 5.96	12.22 ± 7.42	12.64	<0.001	1.10	↓
**BSI-18**							
Anxiety	5.38 ± 4.38	1.65 ± 2.31	9.54	<0.001	0.82	↓
Somatization	3.88 ± 5.24	1.26 ± 1.74	5.84	<0.001	0.51	↓
Depression	3.88 ± 5.24	2.20 ± 3.37	3.69	<0.001	0.32	↓
GSI	13.18 ± 11.77	5.11 ± 6.79	8.09	<0.001	0.70	↓
DLQI	15.34 ± 4.97	0.57 ± 1.44	33.84	<0.001	2.93	↓
**WHOQOL-BREF**							
Physical domain	45.52 ± 14.70	67.24 ± 10.52	14.78	<0.001	−1.28	↑
Psychological domain	28.70 ± 8.79	53.79 ± 10.06	24.36	<0.001	−2.10	↑
Social relations domain	42.11 ± 17.24	62.91 ± 14.67	13.00	<0.001	−1.13	↑
Environment domain	45.84 ± 10.15	60.64 ± 11.37	14.16	<0.001	−1.23	↑

BSI-18, Brief Symptom Inventory-18; DLQI, Dermatology Life Quality Index; GSI, Global Severity Index; PASI, Psoriasis Area and Severity Index; PHQ-9, Patient Health Questionnaire-9; PSS, Perceived Stress Scale; WHOQOL-BREF, World Health Organization Quality of Life – BREF. Higher scores indicate greater symptom severity or perceived stress, except for QoL measures (DLQI, WHOQOL-BREF), where higher scores indicate worse and better quality of life, respectively.

### 3.3 Stepwise regression analyses predicting clinical, psychological and QoL outcomes from baseline variables

Variables included and excluded from each model, along with standardized beta coefficients and significance levels, are reported in Supplementary Table 3. The model predicting depressive symptoms (PHQ-9 at T1) explained 21.3% of the variance [Adjusted R^2^ = 0.213, F(3,125) = 12.519, *p* < 0.001]. Significant predictors included higher baseline PHQ-9 score (β = 0.329, *p* < 0.001), the presence of psychiatric comorbidity (β = 0.257, *p* = 0.001), and lower baseline PASI score (β = −0.191, *p* = 0.017). The model predicting perceived stress (PSS at T1) explained 20.7% of the variance [Adjusted R^2^ = 0.207, F(4,124) = 9.328, *p* < 0.001]. Significant predictors were higher baseline PSS score (β = 0.354, *p* < 0.001), living alone (vs. with others; β = 0.207, *p* = 0.010), no prior biologic therapy (β = −0.171, *p* = 0.032), and lower baseline PASI score (β = −0.198, *p* = 0.013). Global psychological distress (BSI-18 GSI) was predicted by higher baseline somatization (β = 0.258, *p* = 0.025), presence of psychiatric comorbidity (β = 0.234, *p* = 0.004), higher baseline perceived stress (β = 0.181, *p* = 0.041), and lower PASI (β = −0.236, *p* = 0.003), accounting for 22.4% of the variance [Adjusted R^2^ = 0.224, F(4,124) = 10.221, *p* < 0.001]. The anxiety subscale was significantly associated with higher baseline somatization (β = 0.258, *p* = 0.003), female sex (β = 0.170, *p* = 0.039), presence of psychiatric comorbidity (β = 0.197, *p* = 0.022), and lower PASI (β = −0.185, *p* = 0.022), with an Adjusted R^2^ of.214 [F(4,124) = 9.724, *p* < 0.001]. Model-specific regressions confirmed that females reported significantly higher anxiety scores than males (β = 0.260, *p* = 0.003). The somatization subscale was predicted by higher baseline somatization score (β = 0.258, *p* = 0.005), female sex (β = 0.230, *p* = 0.006), and lower environmental QoL at baseline (β = −0.190, *p* = 0.046), with an Adjusted R^2^ = 0.151 [F(3,125) = 8.599, *p* < 0.001]. The depression subscale was predicted by baseline PHQ-9 score (β = 0.298, *p* < 0.001), psychiatric comorbidity (β = 0.275, *p* < 0.001), lower PASI (β = −0.252, *p* = 0.002), and being not employed (vs. employed; β = −0.159, *p* = 0.042), explaining 26.8% of the variance [Adjusted R^2^ = 0.268, F(4,124) = 11.351, *p* < 0.001]. Simple models confirmed the direction of effect: non-employed individuals showed higher depression scores than employed ones, while students did not differ significantly. The model predicting the WHOQOL-BREF physical health domain included economic status as the only significant predictor (β = 0.232, *p* = 0.008), explaining 4.7% of the variance [Adjusted R^2^ = 0.047, F(1,127) = 7.247, *p* = 0.008]. Follow-up models confirmed that higher income was significantly associated with better physical QoL, particularly in the high-income group (β = 0.317, *p* = 0.002), with a smaller effect for middle-income (β = 0.194, *p* = 0.050). The psychological domain of the WHOQOL-BREF was significantly predicted by lower baseline somatization (β = −0.252, *p* = 0.008), lower BMI (β = −0.282, *p* < 0.001), higher baseline physical QoL (β = −0.240, *p* = 0.001), lower perceived stress (β = −0.299, *p* = 0.001), being employed (β = 0.179, *p* = 0.025), and better baseline social relationships (β = 0.213, *p* = 0.026). In separate regressions, students reported significantly higher psychological QoL compared to employed individuals (β = 0.180, *p* = 0.039), whereas non-employed participants did not differ significantly. The model for the WHOQOL-BREF social relationships domain included economic status (β = 0.213, *p* = 0.014) and previous biologic therapy (β = −0.213, *p* = 0.018), with an Adjusted R^2^ of.075 [F(2,126) = 6.185, *p* = 0.003]. Dummy-coded analyses confirmed that both middle-income (β = 0.378, *p* < 0.001) and high-income participants (β = 0.314, *p* = 0.001) reported better social QoL compared to those with low income. Prior biologic therapy was associated with poorer social QoL at follow-up but showed no effect at baseline. Environmental QoL was predicted by lower baseline perceived stress (β = −0.347, *p* < 0.001) and higher economic status (β = 0.213, *p* = 0.010), with an Adjusted R^2^ of.162 [F(2,126) = 13.366, *p* < 0.001]. Additional regressions confirmed stronger effects for high income (β = 0.305, *p* = 0.002) than for middle income (β = 0.267, *p* = 0.007). The DLQI score at follow-up was significantly predicted by marital status (β = 0.261, *p* = 0.003), with an Adjusted R^2^ of.061 [F(1,127) = 9.289, *p* = 0.003]. Regression with dummy variables indicated that only widowed individuals showed higher DLQI scores (β = 0.381, *p* < 0.001) compared to singles, whereas being married/cohabiting or separated/divorced was not associated with dermatology-related QoL.

## 4 Discussion

While biologics are effective in achieving skin clearance, their broader impact on mental health and QoL remains less explored, particularly in real-world routine care. This study adds real-world evidence by assessing a wide range of psychological and HRQoL outcomes in patients with moderate-to-severe psoriasis treated with biologics.

### 4.1 Impact on psychological distress, wellbeing and QoL

Although the clinical efficacy of biologic therapies is extensively supported by randomized controlled trials and large observational cohorts (Armstrong et al., 2022; Warren et al., 2024), participants in this study showed higher rates of clinical response, as proved by the significant reduction in PASI and DLQI score, broadly consistent with the upper range reported in selected real-world and clinical trial populations, especially for IL-17 and IL-23 inhibitors. These results may reflect specific contextual and methodological factors, such as mixed retrospective–prospective data collection, structured follow-up and the characteristics of a tertiary care setting. Beyond skin clearance, study findings highlight the relevant psychosocial benefits of biologic therapies, proved by moderate-to-large effect sizes. These results are consistent with previous studies documenting significant reductions in anxiety and depression and in QoL impairment after 8 weeks of biologic therapy (Liu L. et al., 2023). Similarly, Strober et al. (2018, 2024) found that patients with psoriasis treated with biologic agents had a 17% lower risk of developing depression compared to those receiving conventional systemic therapies (HR = 0.83; 95% CI 0.72–0.97). These observations are further sustained by the systematic review conducted by de Ruiter and Rustemeyer (2022), which aggregated data from over 19,000 patients and concluded that biologics consistently produce clinically meaningful improvements in QoL. Although these external findings support the broader evidence base, it should be noted that our cohort did not include patients treated with conventional systemic therapies; therefore, any references to differences between biologic and conventional treatments in this section are based entirely on previous studies and are reported solely to contextualize the potential clinical and psychosocial relevance of our findings. Improvements in QoL observed in our participants were notable but consistent with previous studies (Iskandar et al., 2017; Pinter et al., 2024). Recent prospective evidence similarly shows that 6 months of biologic therapy yield parallel improvements in PASI, DLQI, depression, and anxiety (Timis et al., 2023). Although less commonly employed in psoriasis research, the WHOQOL-BREF used in this study offers several advantages, particularly its ability to capture systemic and psychosocial dimensions often overlooked by dermatology-specific tools. In this study, all four domains improved significantly from baseline to follow-up, with large effect sizes in physical (*r* = 0.82) and psychological (*r* = 0.86) domains. These findings are in line with previous research (Owczarek and Jaworski, 2016; Frede et al., 2023). Although sex did not significantly predict dermatology-specific or global quality-of-life outcomes, it emerged as an independent predictor of anxiety and somatization symptoms, with female patients reporting higher scores on both dimensions. This partially aligns with previous findings from the CANOVA sex analysis and the PSYCHAE study, which reported greater psychological burden and lower treatment satisfaction in women (Finzi et al., 2007; Colombo et al., 2022), and with evidence showing higher rates of depression among female patients due to the stronger impact of visible lesions on body image(Kouris et al., 2017). Consistently, the Global Psoriasis Atlas identified female sex as an independent predictor of poorer QoL and capability (Silva et al., 2025). While PASI responses appear broadly comparable between sexes, earlier research has frequently documented lower quality-of-life scores in female patients, particularly in DLQI and WHOQOL-BREF domains. Our findings suggest that sex-related differences in psychological symptomatology may not always translate into lower overall QoL scores, possibly reflecting distinct coping styles or reporting patterns. Further research is warranted to clarify how sex influences the subjective experience of disease and treatment response in psoriasis. Study findings confirmed a significant reduction in substantial perceived stress (PSS ≥ 27), from 15.0% at baseline to 1.5% at follow-up. Some evidence suggests that perceived stress may contribute to fatigue, flare frequency, and reduced QoL in psoriasis patients, highlighting a potentially underrecognized dimension in biologic therapy outcomes (Pancar Yuksel et al., 2019; Aktaş Karabay et al., 2020). A recent study by Wannarit et al. (2024) confirmed that depressive symptoms in patients with psoriasis were significantly associated with higher levels of perceived stress, greater psychosocial impact, and lower QoL. Our results add to this emerging body of evidence, suggesting that biologic therapy may reduce systemic stress reactivity, thereby contributing to global psychosocial recovery. Taken together, findings from this study resonate with those of Ntawuyamara et al. (2025), Wu et al. (2024), who reported substantial improvements in dermatology-specific QoL, body image, psychological distress and wellbeing after biologic therapy. These converging results suggest that benefits of biologic therapy extend beyond cutaneous clearance and contribute to holistic recovery across emotional, social, and functional domains.

### 4.2 Predictors of psychological and quality-of-life outcomes

Baseline psychological symptom levels (T0, retrospectively collected) consistently emerged as the strongest predictors of their corresponding follow-up outcomes (T1, prospectively assessed) supporting continuity over time of psychological distress in psoriasis patients previously observed (Liu J. et al., 2023; Strober et al., 2024). Beyond baseline distress, study findings showed the independent contribution of clinical disease severity (i.e., PASI score) to a wide range of psychological outcomes, including depression, anxiety, stress, and somatization, controlling for baseline psychological levels. This supports a biopsychosocial interpretation in which the somatic burden of the disease contributes to emotional dysregulation (Gordon et al., 2018; Wannarit et al., 2024; Wintermann et al., 2024). Although this result primarily reflects the expected continuity between baseline and follow-up measures, it nevertheless underscores the importance of accounting for baseline perceived stress and its trajectory throughout treatment when evaluating psychological outcomes. Sex and psychiatric comorbidity were also identified as significant and independent predictors of anxiety and somatization. These sex-related effects emerged in our multivariable models at follow-up, despite the absence of substantial baseline differences in simple male–female group comparisons (Supplementary Table 5). This pattern suggests that sex may exert its influence primarily through differential psychological trajectories during treatment rather than through pre-treatment symptom severity. Notably, while sex did not significantly predict dermatology-specific or overall QoL outcomes in this study, it independently contributed to anxiety and somatization symptoms in multivariate regression analysis. This may suggest that sex-specific psychological mechanisms may operate independently of general QoL score. Colombo et al. (2022), Wu et al. (2024) similarly reported greater impairment in QoL among female patients, particularly in the domains of daily functioning and interpersonal relationships, although neither study explicitly addressed psychiatric comorbidity. Our data further underscore the independent impact of psychiatric comorbidities on psychological outcomes, supporting the need for integrated care. Notably, this association emerged despite the relatively low prevalence of psychiatric comorbidities in our sample (5.3%), underscoring the strength and clinical relevance of their predictive contribution. This is consistent with Strober et al. (2024), who demonstrated that biologic therapy was associated with a reduced risk of incident depression in psoriasis patients, even after adjusting for multiple psychiatric comorbidities at baseline—including anxiety, substance use disorders, bipolar disorder, and schizophrenia. Additional evidence from Jiang et al. (2023) confirms that psychiatric disorders affect up to 25% of patients with psoriasis, substantially compromising both QoL and treatment adherence. Similarly, Bavière et al. (2020) identified anxiety as the sole independent predictor of mental QoL in patients with psoriatic arthritis, explaining nearly 29% of the variance. These converging findings advocate for sex-sensitive and psychologically integrated management strategies in psoriatic disease. Interestingly, marital status emerged as a significant predictor of dermatology-specific QoL at follow-up, with widowed individuals reporting significantly higher DLQI scores compared to other groups. This may reflect the psychological impact of social isolation or bereavement in the context of chronic inflammatory disease. However, this finding contrasts with results from Wu et al. (2024), where marital status was not associated with QoL or body image outcomes. Such discrepancies may reflect differences in cultural context, sample characteristics, or the use of clinician-reported versus self-reported measures. Furthermore, the history of previous biologic therapy was not associated with perceived stress or social QoL at baseline but emerged as a significant predictor of both outcomes at follow-up. This pattern suggests a potential delayed psychosocial impact of prior treatment exposure, possibly reflecting residual disease burden, treatment fatigue, or demoralization related to previous therapeutic failures. These findings are consistent with data from the CorEvitas registry (Blauvelt et al., 2024), which reported that bio-experienced patients had higher odds of moderate-to-severe DLQI impairment, particularly after treatment switching. Finally, we observed that the WHOQOL-BREF psychological domain was the most significantly predicted QoL outcome, with contributions from multiple psychosocial and clinical factors, including BMI, somatization, baseline QoL scores, perceived stress, and employment status. In addition, economic status emerged as a significant predictor across multiple WHOQOL-BREF domains, with both middle- and high-income patients reporting better physical, social, and environmental QoL compared to those with low income. These results confirm previous findings by Bavière et al. (2020), who found that anxiety was the sole independent predictor of mental QoL in patients with psoriatic arthritis, accounting for nearly 29% of the variance. Altogether, these findings highlight that multidimensional psychosocial determinants — rather than disease severity alone — play a central role in shaping patients’ psychological distress and wellbeing in chronic inflammatory conditions (Bavière et al., 2020). In conclusion, findings from predictive analyses emphasize the importance of integrating clinical, psychological, and sociodemographic variables to understand patient trajectories in moderate-to-severe psoriasis.

## 5 Strengths and limitations

This study offers real-world evidence on the impact of biologic therapy in a cohort of patients with moderate-to-severe plaque psoriasis treated at a tertiary referral center. By assessing clinical, psychological, and QoL outcomes in parallel, it adopts a multidimensional approach that reflects the complexity of psoriasis-related burden. The availability of data both at treatment initiation and at 6-month follow-up enabled the evaluation of short-term therapeutic trajectories in routine clinical practice. Nevertheless, several limitations should be considered. First, the single-center setting in Northern Italy may limit the generalizability of findings to other regions or healthcare systems, and the overall sample size (*N* = 133) may not capture the full heterogeneity of the psoriasis population. As an observational exploratory study, causal inferences patient expectations or lifestyle cannot be established, and improvements may partially reflect unmeasured factors such as changes. Inclusion was restricted to patients completing the 6-month follow-up, which may have introduced selection bias and potentially overestimated treatment efficacy (e.g., high PASI 100 rates). In addition, key psychosocial constructs (e.g., resilience, coping, social support) were not assessed, and the 6-month time frame—although aligned with real-world care—limits insight into long-term outcomes. Regression analyses may also be prone to overfitting, and drug-specific effects could not be examined due to sample size constraints. Finally, reliance on self-reported measures may have introduced bias related to mood state, health literacy, or social desirability. Moreover, some subgroups in our sample were relatively small (e.g., patients achieving PASI 75 or <75, and students), which limits the precision of estimates for these categories and precludes firm conclusions about subgroup-specific effects; therefore, these findings should be interpreted with caution. In addition, because T0 data were collected retrospectively and T1 data prospectively, potential confounding related to differences in data-collection timing cannot be excluded. Although T0 values reflect baseline assessments routinely recorded at biologic initiation, variability in the exact timing and completeness of retrospective documentation may introduce information bias and unmeasured heterogeneity that could not be statistically controlled.

## 6 Conclusion and future directions

This ambispective real-world study highlights the multifaceted benefits of biologic therapy in moderate-to-severe psoriasis, demonstrating improvements not only in skin severity but also in psychological distress and QoL. High clearance rates underscore the influence of therapeutic context, patient selection, and methodological rigor in routine clinical practice. The observed reductions in depression, anxiety, and stress reinforce the need to integrate psychological outcomes into dermatologic care. These findings support a patient-centered approach that acknowledges the interplay between skin disease, mental health, and social vulnerability. Future studies should focus on refining stratification tools, personalizing treatment, and elucidating immuno-psychological mechanisms—pursuing not only skin clearance, but full recovery of wellbeing.

## Data Availability

The raw data supporting the conclusions of this article will be made available by the authors, without undue reservation.
