# Supplementary material for: From skin clearance to psychological wellbeing: real-world outcomes of biologic therapy in psoriasis
Source: Front Psychol. 2026 Feb 13;17:1735777. doi: 10.3389/fpsyg.2026.1735777 (PMC12946107; doi:10.3389/fpsyg.2026.1735777)
Supplement: Supplementary file 1 [file Table_1.docx]

**Table S1. Clinical, psychological, and quality-of-life measures used in the study**

| **Instrument** | **Reference** | **Description** | **No. of items** | **Score range** | **Interpretation** | **Common cut-offs / categories** |
| --- | --- | --- | --- | --- | --- | --- |
| **Psoriasis Area and Severity Index (PASI)** | Fredriksson & Pettersson, 1978 | Composite index evaluating erythema, induration, scaling, and body surface area affected in four regions. | — | 0–72 | Higher = greater psoriasis severity. | PASI 75/90/100 = ≥75%, ≥90%, 100% reduction from baseline. |
| **Patient Health Questionnaire-9 (PHQ-9)** | Spitzer et al., 1999 | Depression screening tool based on DSM-IV criteria, assessing symptom frequency over the last 2 weeks. | 9 | 0–27 | Higher = greater depressive symptom severity. | 0–4 minimal, 5–9 mild, 10–14 moderate, 15–19 moderately severe, 20–27 severe. |
| **Perceived Stress Scale (PSS)** | Cohen et al., 1983 | Measures perceived stress over the last month, focusing on unpredictability, uncontrollability, and overload. | 10 | 0–40 | Higher = greater perceived stress. | 0–13 low, 14–26 moderate, 27–40 high. |
| **Brief Symptom Inventory-18 (BSI-18)** | Derogatis & Melisaratos, 1983 | Self-report measure of psychological distress with three subscales—Depression, Anxiety, and Somatization—and a Global Severity Index (GSI) representing overall psychological distress. | 18 | Subscales: 0–24; GSI: 0–72 | Higher = greater psychological symptom burden. | T-scores ≥ 63 on GSI or on two subscales = clinically significant distress. |
| **Dermatology Life Quality Index (DLQI)** | Finlay & Khan, 1994; Mazzotti et al., 2003, 2005 | Dermatology-specific QoL assessing impact over the last week across symptoms, daily activities, work, relationships, and treatment. | 10 | 0–30 | Higher = worse dermatology-specific QoL. | 0–1 no effect, 2–5 small, 6–10 moderate, 11–20 very large, 21–30 extremely large effect. |
| **World Health Organization Quality of Life – BREF (WHOQoL-BREF)** | THE WHOQOL GROUP, 1998 | Generic QoL tool assessing four domains: Physical Health, Psychological Health, Social Relationships, and Environment. | 26 | Domain scores: ~4–20 (raw) | Higher = better quality of life. | No fixed clinical cut-offs; domain scores can be transformed to 0–100 scale for interpretation. |

**Note.** Abbreviations: BSI-18 = Brief Symptom Inventory-18; DLQI = Dermatology Life Quality Index; GSI = Global Severity Index; PASI = Psoriasis Area and Severity Index; PHQ-9 = Patient Health Questionnaire-9; PSS = Perceived Stress Scale; WHOQOL-BREF = World Health Organization Quality of Life – BREF.

**Table S2. Independent variables included in stepwise multiple linear regression models**

| **Category** | **Variable name** | **Description / Coding** |
| --- | --- | --- |
| **Sociodemographic** | Age | Years (continuous) |
|  | Sex | 0 = male, 1 = female |
|  | Marital status | Dummy-coded: married/cohabiting (ref), single, separated/divorced, widowed |
|  | Employment status | Dummy-coded: employed (ref), unemployed, retired, student |
|  | Education level | Years of education (continuous) or categorical dummy-coded |
|  | Cohabitation status | 0 = living with others (ref), 1 = living alone |
|  | Economic status | Dummy-coded: low (ref), middle, high |
| **Clinical** | Medical comorbidities | 0 = no, 1 = yes (≥1 chronic medical condition) |
|  | Psychiatric comorbidities | 0 = no, 1 = yes (current or past, confirmed at T1) |
|  | BMI | kg/m² (continuous) |
|  | Baseline PASI score | 0–72 |
|  | Prior biologic therapy | 0 = no, 1 = yes |
| **Baseline psychometric measures** | PHQ-9 total score | 0–27 |
|  | PSS total score | 0–40 |
|  | DLQI total score | 0–30 |
|  | BSI-18 depression | 0–24 |
|  | BSI-18 anxiety | 0–24 |
|  | BSI-18 somatization | 0–24 |
|  | WHOQoL-BREF physical domain (%) | 0–100, higher = better QoL |
|  | WHOQoL-BREF psychological domain (%) | 0–100, higher = better QoL |
|  | WHOQoL-BREF social relationships domain (%) | 0–100, higher = better QoL |
|  | WHOQoL-BREF environment domain (%) | 0–100, higher = better QoL |

**Note.** All categorical variables were dummy-coded for inclusion in regression models. Multi-level categorical variables were analyzed with separate dummy variables to compare each level with the reference category.

**Table S3. Standardized beta coefficients (β) from stepwise linear regression models predicting clinical, psychological, and quality-of-life outcomes at six-month follow-up (T1).**

|  | **OUTCOME** **T1** | | | | | | | | | | | |
| --- | --- | --- | --- | --- | --- | --- | --- | --- | --- | --- | --- | --- |
|  | **PASI** | **PHQ-9** | **PSS** | **BSI-18** | | | | **WHOQOL-BREF** | | | | **DLQI** |
|  |  |  |  | *Depression* | *Anxiety* | *Somatization* | *GSI* | *Physical Health domain* | *Psychological domain* | *Social Relationships domain* | *Environment domain* |  |
| **Sociodemographic and clinical predictors** | | | | | | | | | | | | |
| Sex | .071 | .073 | .082 | .058 | **.170^*^** | **.230^*^** | .114 | –.027 | -.121 | –.082 | –.082 | –.057 |
| Age | .077 | .028 | –.020 | -.083 | –.004 | .063 | .025 | –.138 | .109 | –.048 | –.048 | .141 |
| Psoriatic arthritis | –.069 | –.018 | .028 | -.074 | –.035 | –.018 | –.034 | –.059 | -.019 | –.106 | –.106 | –.088 |
| Previous biologic treatment | –.061 | –.093 | **–.171^*^** | -.151 | –.129 | –.032 | –.102 | .060 | .081 | –.001 | –.001 | –.035 |
| Medical comorbidity | –.007 | .058 | –.040 | -.113 | –.043 | –.017 | –.029 | –.059 | -.061 | –.133 | –.133 | .035 |
| Psychiatric comorbidity | –.009 | **.257^*^** | –.001 | **.275^**^** | **.197^*^** | .079 | **.234**^*^ | –.148 | -.137 | –.026 | –.026 | –.068 |
| BMI | .027 | –.012 | –.130 | -.076 | –.069 | .091 | –.058 | –.126 | **-.282^**^** | .020 | .020 | .031 |
| Marital status | **–.298^**^** | –.011 | –.058 | -.057 | .017 | –.011 | –.025 | .078 | .073 | –.006 | –.006 | **.261^**^** |
| Employment status | –.066 | –.143 | –.096 | **-.159^*^** | –.068 | –.079 | –.143 | .052 | **.179^*^** | .084 | .084 | –.086 |
| Living arrangement | –.153 | –.002 | **+.207^*^** | .003 | .034 | .026 | .030 | –.004 | -.122 | –.107 | –.107 | –.128 |
| Educational level | .063 | –.044 | .082 | -.024 | –.069 | –.129 | –.045 | .061 | .014 | –.055 | –.055 | –.103 |
| Economic status | .020 | -.064 | -.013 | -.065 | -.009 | -.113 | -.069 | **.232^*^** | .132 | **.213^*^** | **.213*** | .004 |
| **Skin severity predictor (T0)** | | | | | | | | | | | | |
| PASI | .122 | **–.191^*^** | **–.198^*^** | **-.252^*^** | **–.185^*^** | –.119 | **–.236**^*^ | .083 | -.082 | .076 | .076 | .067 |
| **Baseline psychological predictors (T0)** | | | | | | | | | | | | |
| PHQ-9 | –.080 | **.329^**^** | .043 | **.298^**^** | .039 | .012 | .082 | –.010 | -.146 | –.109 | –.109 | –.042 |
| PSS | .048 | .100 | **.354^**^** | .164 | .130 | .040 | **.181**^*^ | –.101 | **-.299^**^** | **–.347^**^** | **–.347^**^** | –.061 |
| BSI-18 | | | | | | | | | | | | |
| *Anxiety* | –.109 | –.066 | .003 | -.077 | –.159 | –.081 | –.184 | –.019 | .063 | –.100 | –.100 | –.108 |
| *Depression* | –.092 | –.514 | .037 | -.312 | .025 | –.004 | .066 | .004 | -.182 | –.144 | –.144 | –.079 |
| *Somatization* | –.088 | .119 | .032 | .086 | **.258^*^** | .177 | **.189*** | –.063 | **-.252^**^** | –.052 | –.052 | –.139 |
| *GSI* | –.107 | –.041 | .028 | -.041 | –.038 | –.041 | –.009 | –.023 | -.181 | –.125 | –.125 | –.116 |
| WHOQOL-BREF | | | | | | | | | | | | |
| *Physical Health domain* | –.160 | –.078 | .024 | -.035 | –.004 | .051 | –.034 | n.i. | **-.240^**^** | .048 | .048 | –.094 |
| *Psychological domain* | –.088 | –.073 | –.007 | .035 | –.065 | –.070 | –.049 | –.048 | n.i | .098 | .098 | –.082 |
| *Social Relationships domain* | –.076 | .028 | .008 | -.085 | .018 | –.002 | .038 | .094 | -.188^*^ | n.i | .055 | –.027 |
| *Environment domain* | –.013 | –.088 | –.068 | .083 | –.151 | **–.190^*^** | –.137 | .167 | .120 | –.063 | n.i | .037 |
| DLQI | .151 | –.078 | –.020 | -.035 | .126 | –.070 | .052 | –.032 | -.056 | .006 | .006 | .067 |

**Note.** All categorical variables were dummy-coded for inclusion in regression models. Multi-level categorical variables were analyzed with separate dummy variables to compare each level with the reference category. All variables considered in the final models are reported. Values represent standardized regression coefficients (β). p < .05; **p** < .01. Variables not included in the final model are indicated as “n.i.” (not included).
**Reference categories for categorical predictors:** Sex = male; Marital status = single; Employment status = employed; Living arrangement = living alone; Economic status = low; Educational level = no qualification; Previous biologic treatment = no; Psoriatic arthritis = no; Comorbidities = no.
**Abbreviations:** BSI-18 = Brief Symptom Inventory-18; DLQI = Dermatology Life Quality Index; GSI = Global Severity Index; n.i. = not included; PASI = Psoriasis Area and Severity Index; PHQ-9 = Patient Health Questionnaire-9; PSS = Perceived Stress Scale; WHOQOL-BREF = World Health Organization Quality of Life – BREF.

**Table S4. Internal Consistency (Cronbach’s Alpha) of Psychometric Instruments at Baseline (T0)**

| **Instrument** | **Domain / Subscale** | **N. items** | **Cronbach’s α** |
| --- | --- | --- | --- |
| **PHQ-9** | Total score | 9 | **0.94** |
| **PSS-10** | Total score | 10 | **0.79** |
| **BSI-18** | Somatization | 6 | **0.88** |
|  | Depression | 6 | **0.83** |
|  | Anxiety | 6 | **0.86** |
|  | Global Severity Index (GSI) | 18 | **0.95** |
| **DLQI** | Total score | 10 | **0.81** |
| **WHOQOL-BREF** | Physical domain | 7 | **0.69** |
|  | Psychological domain | 6 | **0.68** |
|  | Social Relationships domain | 3 | **0.60** |
|  | Environment domain | 8 | **0.64** |

**Note.** Lower internal consistency values for WHOQOL-BREF domains are expected in clinical samples and reflect the heterogeneous and multidimensional nature of these domains. All symptom scales (PHQ-9, PSS-10, BSI-18, DLQI) demonstrated acceptable to excellent reliability (α = .79–.95).

**Abbreviations:** BSI-18 = Brief Symptom Inventory-18; DLQI = Dermatology Life Quality Index; PASI = Psoriasis Area and Severity Index; PHQ-9 = Patient Health Questionnaire-9; PSS = Perceived Stress Scale; WHOQOL-BREF = World Health Organization Quality of Life – BREF.

**Table S5. Baseline psychometric scores by sex (independent-samples t-tests)**

| **Measure** | **Men (N = 84) Mean ± SD** | **Women (N = 49) Mean ± SD** | **t** | **p** |
| --- | --- | --- | --- | --- |
| **PHQ-9 total score** | 4.96 ± 5.39 | 5.59 ± 6.54 | –0.598 | .551 |
| **BSI-18** | | | | |
| **Anxiety** | 5.29 ± 4.21 | 5.53 ± 4.68 | –0.302 | .764 |
| **Somatization** | 3.54 ± 2.99 | 4.59 ± 3.39 | –1.621 | .109 |
| **Depression** | 3.54 ± 4.72 | 4.47 ± 6.03 | –0.930 | .355 |
| **GSI** | 12.36 ± 10.31 | 14.59 ± 13.81 | –0.984 | .328 |
| **PSS-10 total score** | 19.96 ± 5.98 | 22.06 ± 5.74 | –2.000 | **.048** |
| **DLQI total score** | 15.37 ± 4.97 | 15.29 ± 5.02 | 0.093 | .926 |
| **WHOQOL-BREF** | | | | |
| **Physical** | 45.58 ± 14.18 | 45.41 ± 15.70 | 0.062 | .949 |
| **Psychological** | 27.48 ± 8.73 | 30.78 ± 8.58 | –2.127 | **.036** |
| **Social** | 42.76 ± 17.30 | 40.99 ± 17.25 | 0.571 | .569 |
| **Environment** | 46.39 ± 9.74 | 44.90 ± 10.86 | 0.794 | .429 |

**Note.** Values represent baseline (T0) scores prior to biologic therapy initiation. p-values refer to independent-samples t-tests comparing men vs. women. Significant differences were observed for perceived stress (PSS-10) and psychological well-being (WHOQOL-BREF Psychological domain), with women reporting higher stress and lower psychological QoL. No other sex differences were statistically significant. p < .05.

**Abbreviations:** BSI-18 = Brief Symptom Inventory-18; DLQI = Dermatology Life Quality Index; PASI = Psoriasis Area and Severity Index; PHQ-9 = Patient Health Questionnaire-9; PSS = Perceived Stress Scale; WHOQOL-BREF = World Health Organization Quality of Life – BREF.
